# Supplementary material for: WordCommentsAnalyzer: A windows software tool for qualitative research
Source: F1000Res. 2018 Sep 4;7:536. Originally published 2018 May 3. [Version 2] doi: 10.12688/f1000research.14819.2 (PMC6137411; doi:10.12688/f1000research.14819.2)
Supplement: Supplementary file 3 [file f1000research-7-17614-s0002.tgz › b2a79496-4ada-47c4-9682-a99ce867836d.zip!/Eight Iranian Health Journals/Archives of Rehabilitation_38.docx]

© Archives of Rehabilitation. This work is licensed under CC BY-NC 4.0 (https://creativecommons.org/licenses/by-nc/4.0/).

Volume & Issue: دوره 11 - شماره 2

Title: مقایسه کینماتیک کتف بین زنان مبتلا به نشانگان شلی مفصلی عمومی و زنان سالم در حین بالا بردن بازو

http://rehabilitationj.uswr.ac.ir/article-1-476-fa.html

هدف: یکی از مشکلات افراد مبتلا به نشانگان شلی مفصلی عمومی دررفتگی و نیمه دررفتگی‌های حاد و مکرر شانه می‌باشد. با توجه به اهمیت کتف در ثبات و عملکرد شانه، هدف این مطالعه بررسی مقایسه ای وضعیت و راستای سه بعدی کتف در زنان مبتلا به پرتحرکی و شلی مفصلی و زنان سالم در حین بالا بردن بازو در صفحات فرونتال و ساژیتال می باشد. روش بررسی: در این مطالعه مقطعی و مورد– شاهدی، &zwj۱۶ زن دچار شلی مفصلی به روش ساده و در دسترس و 16 زن سالم به روش همتاسازی و جورکردن با گروه مورد بر اساس سن، شاخص توده بدن و دوره قاعدگی انتخاب و با استفاده از دستگاه آنالیز حرکتی ویکون وی ۴۶۰، جهت قرارگیری ( زوایای چرخش رو به بالا، تیلت خلفی، چرخش داخلی) و وضعیت کتف (بالا، پایین و داخل، خارج) آنها در زوایای مختلف بالا بردن بازو مورد سنجش و مقایسه قرار گرفت. جهت تحلیل داده‌های حاصل از آزمون تی مستقل استفاده گردید. یافته‌ها: در زوایای۹۰ درجه (۰/۰۳=P)، ۱۲۰ درجه (۰/۰۱=P) و دامنه کامل حرکتی شانه (۰/۰۴=P)، میزان چرخش رو به بالای اسکاپولا در صفحه ساژیتال در گروه مورد نسبت به گروه شاهد کاهش داشت. همچنین میزان جابجایی خارجی در صفحه ساژیتال در زوایای ۹۰ درجه (۰/۰۲=P) و ۱۸۰ درجه (۰/۰۲=P) و در صفحه فرونتال در زوایای ۱۲۰ درجه (۰/۰۲=P) و ۱۸۰ درجه (۰/۰۱=P) کاهش معناداری را در گروه بیمار نسبت به گروه سالم نشان داد. نتیجه‌گیری: تغییر کینماتیک کتف یکی از عوامل مساعد کننده بروز آسیبهای شانه در زنان مبتلا به شلی مفصلی بوده و شاید نقص در سیستم عصبی– عضلانی عامل بوجود آورنده تغییرات در کینماتیک استخوان کتف در افراد مبتلا به نشانگان شلی مفصلی عمومی باشد.

Title: تأثیر ورزش هوازی بر نگرش زنان در مورد تصویر بدنی

http://rehabilitationj.uswr.ac.ir/article-1-477-fa.html

هدف: فشارهای اجتماعی نشأت گرفته از افزایش علاقمندی به لاغری و نظرات گسترده در مورد ساختار بدن باعث شده است که نارضایتی از بدن در میان زنان که می تواند منشاء کاهش عزت نفس، افسردگی و سایر اختلالات باشد، بسیار شایع شود. هدف از انجام این مطالعه، بررسی تأثیر ورزش هوازی کوتاه مدت بر جنبه های مختلف نگرش تصویر بدنی در زنان بزرگسال می باشد. روش بررسی: در این پژوهش مداخله ای و شبه تجربی، از طریق نمونه گیری در دسترس، 82 نفر از زنان بزرگسال (18 تا ۴۵ سال)، از میان 150 نفر از مراجعان به باشگاه های گل و انقلاب شهر تهران با توجه به همسان سازی و در نظر داشتن معیارهای ورود و خروج (عدم شرکت در انواع ورزش در3 ماه گذشته، عدم ابتلا به اختلالات جسمی و وجود نگرش منفی در مورد تصویر بدنی) انتخاب شده و به صورت تصادفی تعادلی به دو گروه مداخله و کنترل تخصیص یافتند. جمع آوری داده ها با استفاده از پرسشنامه جمعیت‌شناختی و پرسشنامه چند بعدی نگرش فرد در مورد بدن خود (MBSRQ) انجام شد. گروه مداخله به مدت 4 هفته و هفته ای 3 روز به مدت 1 ساعت در ورزش هوازی شرکت کردند. داده های حاصل با آزمون های تی مستقل و زوجی و مجذور خی مورد تجزیه و تحلیل قرار گرفت. یافته ها: مقایسه دو گروه بعد از مداخله، حاکی از وجود اختلاف معنادار بین آنها در خرده مقیاس های ارزیابی از ظاهر (P<0.001)، آگاهی از ظاهر (P<0.001)، آگاهی از احساس سلامت جسمانی (P=0.003) و آگاهی از احساس بیماری (P=0.002) بود، ولی در مورد خرده مقیاس های ارزیابی از تناسب جسمانی (P=0.141)، آگاهی از تناسب جسمانی (P=0.888)، ارزیابی از احساس سلامت جسمانی (P=0.072)، رضایت از بخشهای مختلف بدن (P=0.082)، مشغولیت فکری با اضافه وزن (P=0.167) و ارزیابی وزن (P=0.156) بین دو گروه اختلاف معناداری وجود نداشت. نتیجه گیری: دوره های کوتاه مدت ورزش هوازی در بهبود برخی ابعاد نگرش تصویر بدنی در زنان بزرگسال مؤثر بوده و می تواند به عنوان یک ابزار درمانی در کاردرمانی مورد استفاده قرار گیرد.

Title: ارتباط ترس از حرکت و شدت درد با ناتوانی جسمی در بیماران مبتلا به کمردرد مزمن

http://rehabilitationj.uswr.ac.ir/article-1-478-fa.html

هدف: شواهد روزافزونی وجود دارد که در بیماران مبتلا به کمردرد مزمن، ترس از حرکت می تواند به اندازه شدت درد ناتوان کننده باشد. این مطالعه با هدف بررسی رابطه بین ترس از حرکت و شدت درد با ناتوانی جسمی در بیماران مبتلا به کمردرد مزمن و تأثیر عواملی چون سابقه کمردرد، سن و جنسیت بر این ارتباط صورت گرفت. روش بررسی: در این تحقیق تحلیلی و مقطعی، ۱۹۴ بیمار با سابقه کمردرد مزمن بیش از ۳ ماه، که در یک دوره چهار ماهه به هفت کلینیک شهر رشت مراجعه کرده بودند، به روش تمام شمار انتخاب و با استفاده از مقیاسهای درجه‌بندی عددی شدت درد، ناتوانی جسمانی رولند و موریس، ترس ازحرکت تمپا و پرسشنامه مشخصات جمعیت‌شناختی، اطلاعات لازم از آنها اخذ و با محاسبه ضریب همبستگی پیرسون و رگرسیون چند متغیره، تحت تجزیه و تحلیل قرار گرفت. یافته‌ها: ارتباط معناداری بین ناتوانی با ترس از حرکت، شدت درد و سن (۰/۰۰۱>P) و همچنین مدت ابتلا به کمردرد (۰/۰۳۶=P) وجود داشت که البته این روابط بین زنان و مردان متفاوت نبود. در مدل رگرسیون چند متغیره اجرا شده، ترس از حرکت و شدت درد، پیش‌بینی‌کننده‌هایی قوی برای ناتوانی بوده و تفاوتی بین آنها یافت نشد. سپس مدل پیش بینی با افزودن متغیرهای سن، جنسیت و مدت ابتلا انجام شد که ترس از حرکت و شدت درد در پیش بینی ناتوانی برتر از سایر متغیرها بودند. در میان ویژگیهای جمعیت‌شناختی تنها سن دارای ضریب بتای معناداری در پیش‌بینی ناتوانی جسمی بود (۰/۰۰۲=P). نتیجه‌گیری: ترس از حرکت، شدت درد و سن از عوامل مهم و مؤثر بر میزان ناتوانی ادراک شده در افراد مبتلا به کمردرد مزمن هستند. بنابراین جهت به حداقل رساندن ناتوانی، علاوه بر کاهش شدت درد، می توان به بیماران دچار ترس از حرکت، روش های درمانی را که بر کاهش این ترس تمرکز دارند پیشنهاد کرد.

Title: بررسی پایایی آزمون پوردوپگبورد در مرحله خاموش دارویی بیماران پارکینسون

http://rehabilitationj.uswr.ac.ir/article-1-479-fa.html

هدف: بیماری پارکینسون یک اختلال مضمحل کننده اعصاب پیشرونده و شایع بوده که در اواخر سنین بزرگسالی شروع شده و شیوع آن در میانگین سنی 55 تا ۶۰ سال افزایش می یابد. هدف از این مطالعه تعیین پایایی آزمون- بازآزمون و اثر تمرین آزمون پوردوپگبورد (که در سنجش مهارت دستی و ارزیابی‌های توانبخشی به کار می‌رود) در افراد مبتلا به بیماری پارکینسون می باشد. روش بررسی: در این مطالعه تحلیلی که از نوع اعتبارسنجی و روش شناختی است، 24 بیمار مبتلا به پارکینسون بین 50 تا 89 سال به صورت نمونه گیری ساده و در دسترس از درمانگاه های توانبخشی و بیمارستانهای شهرتهران انتخاب شدند. با استفاده از یک پرسشنامه جمعیت‌شناختی، اطلاعات عمومی بیماران اخذ و سپس به منظور تعیین پایایی آزمون پوردوپگبورد، هر خرده آزمون آن سه بار انجام شد و بازآزمون یک هفته بعد تکرار شد. داده‌های حاصل از طریق محاسبه ضریب همبستگی درون گروهی و خطای استاندارد اندازه‌گیری و آزمون آماری تی زوجی مورد تحلیل قرار گرفت. یافته ها: میزان ضریب همبستگی درون گروهی، در مرحله خاموش دارویی در یک آزمون و میانگین آزمون ها، همگی بالای 90/0 بود که حاکی از تکرارپذیری نسبی مطلوب آزمون می‌باشد. میزان خطای استاندارد اندازه‌گیری در تمامی موارد در یک بار آزمون و سه بار آزمون کمتر از 10 درصد حداکثر نمرات کسب شده در آزمون بوده و نشان‌دهنده مطلوب بودن تکرارپذیری مطلق آزمون است. نتیجه گیری: نتایج مطالعه حاضر نشان می دهد که آزمون پوردوپگبورد در ارزیابی مهارت دستی بیماران پارکینسون در مرحله خاموش (off) دارویی به اندازه کافی پایا می باشد.

Title: مقایسه تأثیر رویکرد لباس فضایی و بوبات بر عملکرد حرکتی درشت کودکان مبتلا به فلج مغزی

http://rehabilitationj.uswr.ac.ir/article-1-480-fa.html

هدف: این مطالعه به منظور مقایسه تأثیر رویکرد لباس فضایی با رویکرد بوبات بر عملکرد حرکتی درشت کودکان فلج مغزی 4 تا 11 ساله شهر تهران انجام گرفت. روش بررسی: در این مطالعه تجربی و کارآزمایی بالینی تصادفی، 24 کودک مبتلا به فلج مغزی براساس معیارهای تحقیق، از بین مراجعان مؤسسه توانبخشی ولی‌عصر به‌طور ساده انتخاب و پس از همتا سازی بر اساس سن و سیستم طبقه بندی عملکرد حرکتی درشت، به‌صورت تصادفی ساده به دو گروه لباس فضایی و بوبات تخصیص یافتند. دوره درمانی برای هر دو گروه 36 جلسه، 3 بار در هفته بود. ابزار مورد استفاده، آزمون اندازه گیری مهارتهای حرکتی درشت بود. در تجزیه و تحلیل آماری از آزمون های کولموگروف اسمیرونوف، تی مستقل و تحلیل واریانس برای اندازه های تکراری استفاده شد. یافته ها: هر دو گروه بعد از مداخله، پیشرفت معناداری در مهارتهای حرکتی درشت داشتند (۰/۰۰۱>P). پس از قطع مداخله، گروه لباس فضایی کماکان پیشرفت معناداری داشت (۰/۰۰۷=P)، اما گروه بوبات به‌طور معناداری پسرفت نمود (۰/۰۴=P). بلافاصله پس از مداخله، تفاوت معناداری بین دو رویکرد دیده نشد (۰/۵۹۸=P)، ولی پس از قطع درمان، اختلاف دو رویکرد معنادار بود (۰/۰۰۲=P). نتیجه گیری: هر دو رویکرد لباس فضایی و بوبات در بهبود عملکرد حرکتی درشت کودکان مبتلا به فلج مغزی مؤثرند، ولی با قطع جلسات درمانی، در رویکرد لباس فضایی پیشرفت ادامه یافته، در حالی که در رویکرد بوبات، بهبودی حاصل شده تا اندازه ای از دست می‌رود.

Title: ارتباط بین تفسیر انتقال با کاهش شدت افسردگی در روان درمانی روان‌پویشی کوتاه مدت

http://rehabilitationj.uswr.ac.ir/article-1-481-fa.html

هدف: تفسیر انتقال در روان‌درمانی روان‌پویشی کوتاه مدت تکنیک مهمی به شمار می‌رود. هدف این پژوهش بررسی نقش تفسیر انتقال در پیامد شدت افسردگی 6 بیمار مبتلا به افسردگی اساسی می‌باشد که با روان‌درمانی روان‌پویشی کوتاه مدت تحت درمان قرار گرفته‌اند. روش بررسی: در پژوهش حاضر با استفاده از طرح آزمایشی تک آزمودنی، 6 بیمار مبتلا به افسردگی اساسی که به روش در دسترس انتخاب شده بودند، مورد بررسی قرار گرفتند. شدت افسردگی از طریق پرسشنامه افسردگی بک 3 بار قبل از درمان، 20 بار در دوره درمان بعد از هر جلسه و 4 بار در دوره پیگیری سنجیده شد. داده های تحقیق با استفاده از روش تحلیل میزان اثر (d کوهن) و نمودار به‌طور چشمی مورد تحلیل قرار گرفت. یافته ها: مقایسه تغییرات میانگین نمرات افسردگی دوره درمان با دوره قبل از درمان از طریق تحلیل میزان اثر نشان داد که از 6 بیمار مورد بررسی، 5 نفر کاهش شدت افسردگی از حد متوسط به خفیف داشته‌اند. میزان اثر تغییر شدت افسردگی در دوره درمان در مقایسه با دوره قبل از درمان، در 4 مورد معادل میزان زیاد و 1 مورد معادل میزان متوسط بود. یکی از بیماران در دوره درمان در شدت افسردگی کاهش نشان نداد. تحلیل روند تغییرات از طریق نمودار حاکی از رخداد کاهش پایدار در نیمه دوم درمان بود. نتیجه گیری: در تفسیر انتقال، تمرکز بر روی یک الگوی غالب رابطه بین فردی، در کاهش علائم افسردگی در چارچوب روان‌درمانی تحلیلی کوتاه مدت مؤثر می‌باشد.

Title: بررسی الگوی فعالیت عضلانی کمربند شانه ای زنان در برخی تمرینات انتخابی رایج فیزیوتراپی اندام فوقانی

http://rehabilitationj.uswr.ac.ir/article-1-484-fa.html

هدف: هدف از این مطالعه بررسی الکترومیوگرافیک الگوی فعالیت عضلانی عضلات کمربند شانه ای خانم‌ها در برخی تمرینات عملکردی انتخابی اندام فوقانی که در فیزیوتراپی رایج می باشند بود. روش بررسی: این تحقیق به‌صورت شبه تجربی بر روی یک گروه 36 نفری از زنان سالم که به صورت در دسترس و به روش نمونه گیری ساده انتخاب شده بودند انجام شد. جهت ثبت فعالیت الکتریکی، الکترودهای سطحی روی عضلات سینه‌ای بزرگ، ذوزنقه‌ای فوقانی، ذوزنقه‌ای میانی، تحتِ خاری، سه‌گوش قدامی، سه‌گوش میانی و سه‌گوش خلفی قرار گرفتند. از افراد خواسته شد 6 تمرین عملکردی را که شامل تمرینات دور کردن بازو در دو صفحه فرونتال و اسکاپولار، دی ۲ اف، دی ۲ ای، تری‌پاد و پرس آپ بودند، به‌طور تصادفی انجام دهند. الگوی فعالیت عضلانی در دو حوزه زمانی، زمان شروع به فعالیت و زمان به حداکثر رسیدن فعالیت و حوزه سطح فعالیت، با استفاده از شاخص الکترومیوگرافی یکپارچه نرمال شده (NIEMG) بررسی شد. روش آماری استفاده شده در این تحقیق تحلیل واریانس برای اندازه‌های تکراری بود. یافته‌ها: عضله ذوزنقه‌ای فوقانی در اغلب قریب به اتفاق الگوهای حرکتی مورد بررسی سریعترین عضله بکار گرفته شده و با حداکثر سطح فعالیت در مقایسه با سایر عضلات بود. رفتار عضله سینه‌ای بزرگ در هر دو حوزه زمان و سطح فعالیت وابسته به الگوی فعالیت انجام شده متغیر بود. همچنین فعالیت عضلات سه‌گوش قدامی و خلفی در برخی از الگوهای حرکتی خصوصاً الگوهای حرکتی مایل مانند الگوی حرکتی دور کردن بازو در صفحه اسکاپولار و یا محوری مانند الگوهای حرکتی دی ۲ اف و دی ۲ ای سطح فعالیت بالایی داشت. عضله سه‌گوش میانی در کلیه الگوهای حرکتی به عنوان یک عضله حرکت دهنده اصلی عمل کرده و ذوزنقه‌ای میانی و تحتِ خاری هم تقریباً در کلیه الگوهای حرکتی رفتار تقریباً مشابهی از خود نشان می دادند. نتیجه‌گیری: رفتار عضله ذوزنقه‌ای فوقانی وابسته به شکل الگوی حرکتی نمی باشد، در حالی که عضله سینه‌ای بزرگ وابسته به الگوی حرکتی می باشد. فعالیت عضلات سه‌گوش قدامی و خلفی وابسته به راستای انجام الگوی حرکتی و سه‌گوش میانی وابسته به جابجایی مفصل هدف در الگوی حرکتی می باشد و عضلات ذوزنقه‌ای میانی و تحتِ خاری نیز به‌عنوان عضلات ثبات دهنده – هدایت کننده وابستگی به الگوی فعالیت عضلانی ندارند.

Title: نیمرخ رشد زبان دانش آموزان با و بدون اختلال نارسایی توجه، بیش فعالی

http://rehabilitationj.uswr.ac.ir/article-1-485-fa.html

هدف: هدف تحقیق حاضر بررسی نیمرخ رشد زبان دانش آموزان با و بدون اختلال نارسایی توجه/ بیش فعالی است. روش بررسی: در این پژوهش مقایسه‌ای مورد– شاهدی، از بین دانش آموزان پایه اول ابتدایی شهر قزوین ۳۰ کودک (15 پسر و 15 دختر) با اختلال نارسایی توجه/ بیش فعالی با نمونه‌گیری ساده و ۳۰ کودک سالم که با گروه مورد همتاسازی شده بودند شرکت کردند. ابزارهای به‌کار رفته، آزمون رشد زبان (TOLD-P:3) برای ارزیابی زبان و گفتار، مقیاس درجه بندی کانرز برای معلمان به‌منظور ارزیابی اختلال نارسایی توجه/ بیش فعالی و آزمون ریون برای ارزیابی هوش بودند. داده‌های حاصل با روش تحلیل واریانس مورد بررسی قرار گرفت. یافته ها: بین نیمرخ رشد زبان دانش آموزان دارای اختلال نارسایی توجه/ بیش فعالی و دانش‌آموزان سالم تفاوت معناداری وجود داشت (001/0P<)، اما در بین گروه های جنسی در هر یک از گروه‌های مورد (۰/۲۵۴=P) و شاهد (۱/۰۰=P) تفاوت معنادار آماری وجود نداشت. واژگان تصویری، واژگان ربطی، واژگان شفاهی، درک دستوری، تمایزگذاری کلمه، گوش کردن، سازماندهی، صحبت کردن، معنی شناسی و نحو در گروه مورد به طور معناداری پایین تر از گروه شاهد بود، اما در تقلید جمله، تحلیل واجی و تولید کلمه بین دو گروه تفاوتی وجود نداشت. نتیجه گیری: کودکان با نارسایی توجه/ بیش فعالی در برخی از جنبه های رشد زبان از سطح پایین تری نسبت به کودکان طبیعی برخوردارند.

Title: بررسی تأثیر تقلید حرکتی غیر گفتاری بر توانایی نامیدن در کودکان مبتلا به درخودماندگی(اوتیسم)

http://rehabilitationj.uswr.ac.ir/article-1-486-fa.html

هدف: هدف از این پژوهش، بررسی رابطه تقلید حرکتی غیر گفتاری با توانایی نامیدن و تأثیر تمرینات تقلید حرکتی غیر گفتاری بر افزایش توانایی نامیدن در کودکان مبتلا به درخودماندگی (اوتیسم) است. روش بررسی: در مرحله نخست این مطالعه که به روش مقایسه ای صورت پذیرفت، 22 کودک درخودمانده و 22 کودک سالم با نمونه گیری در دسترس انتخاب و توانایی نامیدن و تقلید حرکتی غیر گفتاری آنها مورد سنجش و مقایسه قرار گرفت. در مرحله دوم، مطالعه به صورت تجربی و مداخله ای صورت گرفت. کودکان درخودمانده به طور تصادفی به دو گروه آزمایشی و کنترل تقسیم شدند. سپس کودکان گروه آزمایش به مدت 60 روز، روزانه یک ساعت تحت تمرین تقلید غیر گفتاری قرار گرفتند. در این مدت گروه کنترل از آموزشهای معمول قبلی بهره گرفتند. قبل و بعد از مداخله، توانایی نامیدن در دو گروه با استفاده از آزمون نامیدن سنجیده شد. در تحلیل داده ها از آزمون تی مستقل و بررسی ضریب همبستگی بهره گرفته شد. یافته ها: تفاوت معناداری در توانایی نامیدن کودکان درخودمانده و سالم دیده شد(P<0.001). همچنین یافته ها حاکی است بین توانایی نامیدن و توانایی تقلید کلامی در کودکان درخودمانده همبستگی مثبت وجود دارد (r=0/878). به علاوه، داده ها حاکی از وجود تفاوت معنادار بین توانایی نامیدن دو گروه آزمایشی و کنترل پس از انجام مداخله می باشد (P<0.001). نتیجه گیری: بین توانایی نامیدن و تقلید حرکتی غیر گفتاری همبستگی مثبت و قوی وجود دارد و انجام تمرینات تقلید حرکتی غیرگفتاری به افزایش توانایی نامیدن در کودکان درخودمانده منجر می گردد.
